# Non-coding RNAs in bladder cancer, a bridge between gut microbiota and host?

**DOI:** 10.3389/fimmu.2024.1482765

**Published:** 2024-11-19

**Authors:** Jun Zou, Baisheng Xu, Peiyue Luo, Tao Chen, Huanglin Duan

**Affiliations:** ^1^ Department of Otorhinolaryngology, The Affiliated Fengcheng Hospital of Yichun University, Fengcheng, Jiangxi, China; ^2^ Department of Urology, The First People's Hospital of Xiushui, Jiujiang, Jiangxi, China; ^3^ The First Clinical College, Gannan Medical University, Ganzhou, Jiangxi, China

**Keywords:** non-coding RNA, gut microbiota, bladder cancer, inflammation, autophagy

## Abstract

In recent years, the role of gut microbiota (GM) in bladder cancer has attracted significant attention. Research indicates that GM not only contributes to bladder carcinogenesis but also influences the efficacy of adjuvant therapies for bladder cancer. Despite this, interventions targeting GM have not been widely employed in the prevention and treatment of bladder cancer, mainly due to the incomplete understanding of the complex interactions between the host and gut flora. Simultaneously, aberrantly expressed non-coding RNAs (ncRNAs) have been frequently associated with bladder cancer, playing crucial roles in processes such as cell proliferation, invasion, and drug resistance. It is widely known that the regulation of GM-mediated host pathophysiological processes is partly regulated through epigenetic pathways. At the same time, ncRNAs are increasingly regarded as GM signaling molecules involved in GM-mediated epigenetic regulation. Accordingly, this review analyzes the ncRNAs that are closely related to the GM in the context of bladder cancer occurrence and treatment, and summarizes the role of their interaction with the GM in bladder cancer-related phenotypes. The aim is to delineate a regulatory network between GM and ncRNAs and provide a new perspective for the study and prevention of bladder cancer.

## 1 Introduction

Bladder cancer is one of the most prevalent tumors in urological surgery, exhibiting a high incidence globally. In 2018, there were 430,000 new cases and 165,000 deaths worldwide. By 2020, the number of new cases had increased to 573,278 (1, 2). Urothelial carcinoma is the most common pathological type of bladder cancer. Non-muscle invasive bladder cancer (NMIBC) is the most frequently encountered variant in clinical practice and accounts for approximately three-quarters of cases (3). Fortunately, current treatment strategies for NMIBC result in a 5-year survival rate exceeding 90%. Nevertheless, the management of NMIBC is suboptimal, as indicated by recurrence rates ranging from 15% to 61% at one year and from 31% to 78% at five years (4). As a result, patients with NMIBC who have undergone transurethral resection of bladder tumor (TURBT) are subjected to prolonged cystoscopic surveillance, which significantly increases their physical discomfort and economic burden. The high incidence and recurrence rates of bladder cancer underscore the urgent need to clarify its underlying mechanisms and explore potential therapeutic strategies for both prevention and treatment.

The gut microbiome (GM) can be regarded as an independent system within humans and animals, comprising a highly complex array of components. It plays crucial roles in nutrient absorption, vitamin synthesis, bile acid and sterol metabolism, as well as in immune modulation and the maintenance of intestinal homeostasis (5, 6). Under normal circumstances, the intestinal microflora and the host maintain a mutually beneficial relationship. The composition of intestinal bacteria is closely related to a specific environment, which includes pH, oxygen level/redox state, nutrients, and humidity/temperature. Maintaining this environment is closely tied to the interaction between various intestinal microflora and the host (7). Deviation from its normal composition suggests the occurrence or progression of certain diseases, including bladder cancer. Advances in GM sequencing and the application of Mendelian randomization have led to the identification of numerous intestinal microorganisms associated with the progression of bladder cancer. These will be elaborated on in detail later. Overall, the role of GM in bladder cancer has attracted increasing attention. It is noteworthy that as early as 1984, a study (8) had confirmed that chronic bacterial infection of the bladder can lead to bladder epithelial tumor-like changes. Bladder bacterial infection leads to frequent damage and repair of bladder epithelial cells through immune inflammatory responses, thereby increasing the risk of gene mutation and malignant transformation of cells (9). In addition, bacterial infection can also activate NF-κB and other cancer-promoting signaling pathways, further promoting the growth, survival, and metastasis of tumor cells (10). However, with the progression of research, it has been found that the gut and urine microbiota have a certain correlation. For example, GM can influence the bacterial susceptibility of the bladder through the gut-bladder axis (11, 12). There is also evidence (13) that the gastrointestinal tract forms a connection with the urinary microbiome through bacterial migration and blood circulation. It is proposed that the induction of bladder cancer by urinary microorganisms may also be regarded as one aspect related to GM. Overall, the role of GM in bladder cancer has continued to attract increasing attention.

Non-coding RNA (ncRNA) is a class of RNA that does not encode proteins. Its discovery is regarded as an important breakthrough in life sciences (14). The completion of the Human Genome Project (HGP) and the progress of the ENCODE project have revealed that protein-coding genes account for less than 2% of the genome. Meanwhile, approximately 90% of the human genome is transcribed into ncRNA (15, 16). Increasing evidence shows that ncRNA plays crucial roles in cellular development, physiological functions, and disease progression (16, 17). Based on their length and structure, ncRNAs are classified into small ncRNAs, circular RNAs (CircRNA), and long ncRNAs (lncRNA). Small ncRNAs comprise microRNA (miRNA), ribosomal RNA (rRNA), and transfer RNA (tRNA). These molecules act as key regulators in gene expression processes such as protein translation and post-transcriptional silencing (18, 19). Consequently, ncRNAs have been intensively studied for their significant roles in regulating human diseases, especially cancer development (20). Additionally, ncRNAs exhibit superior histocompatibility and better tissue penetration compared to traditional small molecule drugs, rendering them promising candidates for cancer therapeutics (21). The interaction between the host and GM forms a complex and intricate regulatory network. However, research in this field is still in its early stages. Notably, ncRNAs have emerged as important mediators in this communication. The role of ncRNAs in host-microbiome interactions, as well as their influence on intestinal homeostasis and related cancers, is attracting increasing attention. Accordingly, this paper examines the differential expression of various ncRNAs in bladder cancer and their potential impacts on tumor development. Additionally, we study the interactions between ncRNAs and GM and their implications in tumorigenesis. By clarifying these relationships, we aim to provide insights into the prevention and treatment of bladder cancer and identify potential biomarkers.

## 2 Role of gut microbiota in bladder cancer

### 2.1 Gut microbiota and the genesis of bladder cancer

The first intuitive evidence stems from the age of onset of bladder cancer. Data suggest that approximately 75% of newly diagnosed bladder cancer patients are over 65 years old, and around 45% are over 75 years old, classifying it as a disease primarily affecting the elderly (22). Furthermore, the GM undergoes changes with age. Young-derived GM has been demonstrated to restore the health of the elderly by altering the microbial composition of their intestines (23). This leads us to thoroughly question the changes in GM during the occurrence of bladder cancer and its impact on tumors. Several research findings uphold this perspective. A case-control study (24) revealed that bladder cancer patients demonstrated an imbalance in the GM, primarily characterized by a reduction in *Clostridium* cluster XI and *Prevotella*, along with decreased butyric acid concentrations and impaired intestinal barrier integrity. In addition, another study (25) found that a soluble high-fiber diet resulted in delayed tumor growth following irradiation compared to other groups. Moreover, rats on the soluble high-fiber diet showed a significantly higher relative abundance of *Bacteroides*, further corroborating the close relationship between GM and tumor progression. With the continuous progress in GM sequencing and Mendelian randomization research, an increasing number of studies are investigating the differential expression of GM-related genes in bladder cancer patients. A bidirectional Mendelian randomization study (26) identified associations between bladder cancer and several genera, such as *Allisonella*, *Lachnospiraceae*, *Oscillibacter*, *Eubacterium coprostanoligenes* group, *Eubacterium ruminantium* group, *Ruminococcaceae*, and *Senegalimassilia*. Similarly, Wang et al. (27) employed a comparable method and concluded that higher abundances of *Bifidobacterium*, *Actinobacteria*, and *Ruminococcus torques* in the gut are associated with an elevated risk of bladder cancer. However, another study (28) identified that *Lachnospiraceae*, *Desulfovibrionales* (Order), *Eubacterium ruminantium* group, *Olsenella*, and *Ruminococcaceae* have causal impacts on bladder cancer. Conversely, *Bacteroidetes* (Phylum), *Eubacteriumbrachy* group, *Ruminococcaceae*, *Rikenellaceae* (Family), *Lachnospiraceae* group, and *Adlercreutzia* exhibit a protective effect against bladder cancer. The final evidence relates to the preventive effect of sulforaphane on bladder cancer. Research (29) has demonstrated that sulforaphane can restore the disrupted intestinal microbiota in N-butyl-N-(4-hydroxybutyl)-nitrosamine-induced bladder cancer mice, repair physiological damage to the intestinal barrier, reduce inflammation and immune responses, and thus prevent chemical-induced bladder cancer. Overall, various abnormally expressed GMs have been found to be closely related to the development of bladder cancer, potentially serving as both pathogenic and protective factors. Moreover, correcting the GM can significantly reduce the carcinogenic impacts on bladder cancer, indicating that a healthy GM plays a crucial role in reducing the risk of bladder cancer.

### 2.2 Impact of gut microbiota on bladder cancer treatment

Chemotherapy and immunotherapy constitute key treatment modalities for bladder cancer. As emerging therapeutic approaches, they offer the advantage of specifically targeting tumor cells, alleviating symptoms, and prolonging survival when compared with traditional treatments (30). However, their efficacy varies considerably among individuals, being influenced by gene expression and the body’s immune status. Additionally, chemotherapy drug resistance remains a significant challenge that affects treatment outcomes. The role of the GM in bladder cancer is crucial, not only in the disease’s progression but also in modulating the efficacy of chemotherapy and immunotherapy. Evidence supporting this view is provided by studies investigating the relationship between antibiotic use and the survival rates of patients with urothelial carcinoma undergoing chemotherapy and immunotherapy. Data from Ashley et al. (31) indicated that antibiotic use in patients treated with atezolizumab was associated with decreased survival rates. In contrast, no such association was observed in patients receiving chemotherapy alone. This implies that excessive antibiotic use may disrupt the intestinal microbiota and specifically reduce the efficacy of cancer immunotherapy. Additionally, a multi-institutional prospective cohort study examined differences in the GM between bladder cancer patients and healthy adults, as well as variations in GM composition among patients with different responses to chemotherapy. The study (32) found that patients with higher levels of *Bacteroides* had a worse response to neoadjuvant chemotherapy. Similarly, Lukas et al. (33) found that *Bifidobacterium pseudolongum*, *Lactobacillus johnsonii*, and *Olsenella* species significantly increased the efficacy of immune checkpoint inhibitors in mouse models of rectal cancer, bladder cancer, and melanoma. Further research has confirmed that this effect is mediated by adenosine, a metabolite of the GM. Another related study (34) found that GM can influence the levels of ketone bodies, such as 3-hydroxybutyrate (3HB), which in turn significantly impacts the anti-tumor efficacy of PD-1 inhibitors. Additionally, fecal microbiota transplantation and single colonization experiments were performed with *Parabacteroides distasonis* to assess the efficacy of combined treatment with immune checkpoint inhibitors (ICIs). The results indicated that this combination therapy increased the intratumoral density of CD4+ and CD8+ T cells and significantly postponed tumor growth. Transcriptome analysis further revealed that the inclusion of *Parabacteroides distasonis* significantly upregulated various pathways related to anti-tumor immune responses (35).

The aforementioned evidence indicates that the GM is closely associated with the onset, progression, and treatment of bladder cancer. Bladder cancer development is often accompanied by dysbiosis. Correcting the GM can not only prevent bladder cancer but also improve the effectiveness of immunotherapy. However, understanding how bladder cancer progression influences changes in the GM and the mechanisms by which the GM modulates treatment efficacy remains a critical issue. It is well-known that the interaction between the host and the GM is crucial for maintaining intestinal homeostasis and immune responses. Furthermore, epigenetic regulation is regarded as a significant mechanism through which the microbiota can modify host physiology. Host-intestinal microbiota communication is essential for understanding the relationship between the GM and bladder cancer. Increasing evidence (36, 37) indicates that ncRNAs serve as a significant communication medium between the host and the intestinal microbiota, influencing the host’s pathophysiological processes. The role of ncRNAs in host-microbiome interactions, as well as their influence on intestinal homeostasis and related cancers, is attracting considerable attention. Accordingly, we have examined the differential expression of various non-coding RNAs in bladder cancer and their potential impacts on tumor progression. Additionally, we have explored the interactions between non-coding RNAs and the GM, and their roles in tumor development. Clarifying these relationships offers valuable insights for bladder cancer prevention, treatment, and the identification of potential biomarkers.

## 3 Another perspective, ncRNA in bladder cancer

### 3.1 miRNA

MiRNA was discovered in 1993. It is a single-stranded ncRNA with a length of approximately 20–22 nucleotides. MicroRNAs primarily regulate gene expression post-transcriptionally in various organisms, exerting an influence on processes such as cell growth, proliferation, differentiation, and apoptosis (38, 39). It is estimated that microRNAs regulate approximately 60% of the human transcriptome (40). The primary microRNA (pri-miRNA) is processed into precursor microRNA (pre-miRNA) by a microprocessor complex comprising the RNase III enzyme Drosha and the double-stranded RNA-binding protein DGCR8. Subsequently, the pre-miRNA is transported by the exportin XPO5, cleaved by the nuclease Dicer, and integrated into the RNA-induced silencing complex (RISC). When the microRNA is complementary to the 3’ untranslated region (3’ UTR) of the target mRNA, the RNA-induced silencing complex (RISC) inhibits protein translation and promotes mRNA degradation by endonucleases (38, 41, 42). Thus, microRNA plays a crucial role in regulating post-transcriptional gene silencing. As a novel tumor marker and therapeutic target, microRNA has attracted significant attention. Research indicates that microRNAs are crucial in tumor initiation, progression, and metastasis. Certain microRNAs function as tumor suppressors by downregulating oncogene expression, thereby inhibiting tumor growth and proliferation. Conversely, other microRNAs act as oncogenes, promoting tumor cell growth, invasion, and metastasis (43–45). Additionally, microRNAs can influence the tumor microenvironment, modulate interactions between tumor cells and stromal cells, and have an impact on tumor angiogenesis and immune evasion (46, 47). These effects have also been observed in the development of bladder cancer.

In a case-control study (48), 106 bladder urothelial carcinoma tissues were compared with normal tissues, uncovering a significant difference in the expression level of miRNA-124-3p between bladder cancer and normal samples. This indicates that miR-124-3p is closely related to bladder cancer and has potential as a predictive marker. Similarly, Yang et al. (49) examined the differential expression of urinary exosomal miRNAs in 116 bladder cancer patients compared to 116 healthy volunteers. Their results revealed elevated levels of urinary exosomal miR-146a-5p, miR-93-5p, miR-663b, miR-21, and miR-4454 in bladder cancer patients. The aforementioned studies demonstrate a strong correlation between miRNAs and bladder cancer. Furthermore, additional *in vivo* and *in vitro* experiments have examined the complex effects of various miRNAs on bladder cancer (**Table 1**). Excessive proliferation is a hallmark of tumor cells and a key driver of cancer progression, involving various complex signaling molecules and pathways, such as growth factors and receptors. miRNAs affect cell proliferation by targeting genes related to these signaling pathways and proteins. In bladder cancer development, several miRNAs (such as miR-12, miR-299-5p) have been discovered to promote bladder cancer cell proliferation by targeting specific genes, such as those regulating the cell cycle and promoting glycolysis (50–52). Furthermore, research (53) has demonstrated that miR-17-5p from urinary exosomes can also promote the growth and invasion of bladder cancer cells by influencing the tumor immune microenvironment. Conversely, some studies (54–65) have shown that certain miRNAs can suppress bladder cancer cell proliferation by downregulating tumor-promoting genes. Tissue invasion and metastasis are additional key characteristics of tumor cells. Various miRNAs have been demonstrated to either promote (50–53) or inhibit (54, 55, 57, 61, 63, 64) tumor cell invasion. A study (59) found that miR-299-3p suppresses bladder cancer progression by downregulating VEGFA levels and inhibiting angiogenesis. Conversely, another study (66) shows that miR-1247-3p, which is highly expressed in tumor-derived exosomes, promotes angiogenesis in bladder cancer. At present, bladder cancer treatment mainly involves surgery and adjuvant chemotherapy. The efficacy of these treatments is significantly affected by tumor cell resistance to chemotherapeutic drugs. Encouragingly, miRNAs have demonstrated potential in enhancing drug resistance in bladder cancer cells. For example, Wu and Yang et al. (57, 60) found that miR-145-5p and miR-15a-5p can respectively counteract chemoresistance mediated by OCT4B and eIF5A2, thereby enhancing the effectiveness of chemotherapy. In general, various miRNAs have been demonstrated to affect bladder cancer cell characteristics by regulating the expression of specific target genes, thereby influencing the progression of the disease. Generally, a large number of miRNAs are differentially expressed in bladder cancer, and they are demonstrated to target genes closely related to the proliferation and invasion of bladder cancer. It should be noted that the effect of miRNA on bladder cancer is not always promoting or inhibiting, which depends on the type of miRNA and the stage of tumor development. Moreover, miRNA can also enhance the drug resistance of bladder cancer cells and increase the effectiveness of chemotherapy.

**Table 1:** Overview of miRNA functions in bladder cancer.

miRNA	Expression	Function	Target genes	Reference
miR-320a-3p	Down	Proliferation (+) Metastasis (+)	IGF2BP3	(54)
miR-873-5p	Down	Proliferation (+) Metastasis (+) Apoptosis (−)	GNMT	(55)
miR-30c-5p	Down	Proliferation (+)	PRC1	(56)
miR-129	Up	Proliferation (+) Metastasis (+)	SOX2	(50)
miR-145-5p	Down	Proliferation (+) Metastasis (+) Resistance (+)	OCT4B	(57)
miR-205-3p	Up	Proliferation (−) Metastasis (−)	GLO1	(58)
miR-299-3p	Up	Proliferation (−) Metastasis (−) Angiogenesis (−)	VEGFA	(59)
miR-105-5p	Up	Proliferation (+) Metastasis (+)	GPR12	(51)
miR-17-5p	Up	Proliferation (+) Metastasis (+) Immunosuppression (+)	ARID4B	(53)
miR-15a-5p	Down	Proliferation (+) Resistance (+)	eIFTA2	(60)
miR-152-3p	Up	Proliferation (−) Metastasis (−) Apoptosis (+)	KIF14	(61)
miR-3960	Up	Proliferation (−) Apoptosis (+)	DEXI	(62)
miR-367-3p	Up	Proliferation (−) Metastasis (−)	RAB23	(63)
miR-145-5p	Up	Proliferation (−) Metastasis (−)	ATIC	(64)
miR-299-5p	Up	Proliferation (+) Metastasis (+)	*DOK7*	(52)
miR-1247-3p	Up	Angiogenesis (+) Metastasis (+)	FOXO1	(66)
miR-150-5p	Up	Proliferation (−) Metastasis (−) Apoptosis (+)	N4BP2L1	(65)

' + ' indicates the awareness of ' promotion ', ' - ' indicates the awareness of ' inhibition '.

### 3.2 lncRNA

LncRNA is characterized as RNA with more than 200 nucleotides that does not undergo translation into proteins. Similar to mRNA, the majority of lncRNAs are transcribed by RNA polymerase II (RNA Pol II) and then degraded by exonucleases or processed by exosomes (42, 67). Moreover, certain lncRNAs undergo a maturation process similar to that of most transfer RNAs and are commonly cleaved by the RNase P enzyme (68). In contrast to small RNA molecules, lncRNAs possess longer sequences and complex spatial configurations. Utilizing Watson-Crick base pairing, lncRNAs can adopt secondary or tertiary three-dimensional structures, enabling them to perform RNA-related functions through nucleic acid complementarity and exhibit protein-like functions through their three-dimensional spatial arrangements (69, 70). This complex interplay highlights the diverse and complex role of lncRNAs in regulating gene expression mechanisms. The functional modes of lncRNAs can be broadly classified into four types (signal, decoy, guide, and scaffold) (71, 72). Signal: Some lncRNAs are transcribed in response to specific stimuli and regulate one or more target genes (either in a cis- or trans-acting manner) (73, 74). Guide: lncRNAs can function in a manner similar to molecular chaperones by binding to proteins (usually transcription factors), directing these proteins to specific DNA sequences, and thereby regulating downstream molecular transcription (either in a cis- or trans-acting manner) (75, 76). Decoy: This class of lncRNAs, upon transcription, binds directly to RNAs or transcription factors, thereby suppressing their activity and associated signaling pathways (77, 78). Scaffold: Moreover, lncRNAs can function as a “central platform” to which multiple related transcription factors can attach. Consequently, when multiple signaling pathways are simultaneously activated within a cell or organism, these downstream effector molecules can interact with the same lncRNA. This interaction promotes the convergence and integration of information from different signaling pathways, enabling the cell or organism to rapidly generate feedback and regulate responses to external signals and stimuli (79, 80). Moreover, as research progresses, lncRNAs are being investigated for their increasingly complex roles in transcriptional regulation. This paper also examines their associated functions in bladder cancer.

A meta-analysis investigating the relationship between lncRNA MALAT1 expression and prognosis in bladder cancer found that elevated levels of lncRNA MALAT1 are associated with a poor prognosis and an increased risk of lymph node metastasis in these patients (81). Furthermore, numerous studies have elucidated the significant role of lncRNAs in the development of bladder cancer (**Table 2**). Several lncRNAs, including LINC00839, lncRNA PVT1, LUCAT1, LINC00958, and BCYRN1, exert a positive influence on both the proliferative viability and invasive capabilities of bladder cancer cells. These lncRNAs interact with specific target genes, such as SOX5, enhancing cell viability, reducing apoptosis, and promoting metastasis by facilitating epithelial-mesenchymal transition (EMT) (82–86). Moreover, it has been found that BACH1-IT2, a lncRNA, contributes to the progression of bladder cancer by stabilizing Siglec-15, which in turn suppresses the local tumor immune microenvironment (87). In contrast, some lncRNAs (such as lncRNA-RP11-498C9.13, lncRNA MEG3, PCAT29, MIR4435-2HG) have been demonstrated to significantly inhibit the proliferation and invasion of bladder cancer cells by targeting downstream genes (88–91). Furthermore, lncRNA BCCE4 has been demonstrated to enhance the anti-tumor immune response and suppress bladder tumorigenesis by potentiating the PD-L1/PD-1 interaction in smoking-associated bladder cancer (92). Distant metastasis is a major cause of mortality in bladder cancer patients. Those with high malignancy are particularly susceptible to lymphatic and bloodstream metastases. Li et al. (93) identified a correlation between LINC00665 and both lymph node metastasis and poor prognosis in bladder cancer patients. Subsequent research revealed that LINC00665 enhances RAB27B expression, thereby inducing a RAB27B-HGF-c-Myc positive feedback loop that promotes lymphangiogenesis and lymph node metastasis in bladder cancer. In addition, lncRNAs promoting blood metastasis have also been reported. BLCa-associated transcript 3 (BLACAT3), an abnormally up-regulated lncRNA in bladder cancer, was found to promote blood metastasis by enhancing bladder cancer angiogenesis (94). Similar to miRNAs, lncRNAs have been implicated in inducing drug resistance in bladder cancer cells. Specifically, LINC00839 regulates SOX5 either directly or indirectly through the miR-142 axis, which increases the resistance of bladder cancer cells to gemcitabine by promoting autophagy (82). Similar to miRNA, many differentially expressed lncRNAs have been identified in bladder cancer, which play an important role in regulating tumor proliferation, invasion, and drug resistance. It is noteworthy that in addition to these conventional tumor phenotypes, some differentially expressed lncRNAs have been found to be associated with blood and lymphatic metastasis of bladder cancer, suggesting that these lncRNAs can be not only used as intervention targets to prevent tumor progression but also serve as potential prognostic markers.

**Table 2:** Overview of lncRNA functions in bladder cancer.

lncRNA	Expression	Function	Target genes	Reference
LINC00665	Up	Lymphangiogenesis (+) Metastasis (+)	RAB27B	(93)
LINC00839	Up	Proliferation (+) Metastasis (+) Resistance (+)	SOX5	(82)
BACH1-IT2	Up	Immunosuppression (+)	Siglec-15	(87)
lncRNA-RP11-498C9.13	Down	Proliferation (−) Metastasis (−)	PYCR1	(88)
LncRNA PVT1	Up	Proliferation (+) Metastasis (+)	STAT5B	(83)
lncRNA MEG3	Up	Proliferation (−) Metastasis (−) Apoptosis (+)	SPRY2	(89)
LUCAT1	Up	Proliferation (+) Metastasis (+) Ferroptosis (−)	IGF2BP1	(84)
PCAT29	Up	Proliferation (−) Metastasis (−)	SCARA5	(90)
LncRNA BCCE4	Up	Immunosuppression (−)	USP18	(92)
BLACAT3	Up	Angiogenesis (+) Metastasis (+)	NCF2	(94)
LINC00958/BLACAT2	Up	Proliferation (+) Metastasis (+)	SAPK	(85)
BCYRN1	Down	Proliferation (+) Metastasis (+) Apoptosis (−)	Not mentioned	(86)
MIR4435-2HG	Up	Proliferation (+) Metastasis (+)	BRCA2 and CCND1	(91)

' + ' indicates the awareness of ' promotion ', ' - ' indicates the awareness of ' inhibition '.

### 3.3 circRNA

CircRNAs represent a subclass of noncoding RNA molecules lacking a 5’ terminal cap and a 3’ terminal poly(A) tail. They form a covalently bonded ring structure through the variable shearing of a distinctively terminally reverse-complementary precursor mRNA, undergoing head-to-tail cyclization (95). This distinctive loop configuration endows enhanced stability compared to linear RNAs. Despite their typically low expression levels, the remarkable durability of circRNAs enables cellular accumulation to levels exceeding those of corresponding linear RNAs (96–98). Among their diverse functions, circRNAs are prominently acknowledged for their involvement in miRNA sponging. CircRNAs sequester miRNAs by binding to complementary sequences, thereby hindering miRNAs from targeting mRNAs. This liberation of mRNAs from miRNA inhibition enables subsequent ribosomal binding and eventual protein translation (99). Furthermore, circRNAs have been reported to modulate alternative splicing or transcription processes and regulate the gene expression of their parental genes by inhibiting transcription start sites (100). Ongoing investigations are continuously uncovering additional functions of circRNAs. With an expanding set of endogenous circRNAs linked to cancer, nonetheless, many of these molecules remain unexplored with regard to their functional significance (101).

Among studies on the roles of circRNAs in bladder cancer, the most extensive research centers on circRNAs functioning as miRNA sponges (**Table 3**). Since various miRNAs are known to regulate cell viability and invasive potential in bladder cancer, it is reasonable to deduce that numerous circRNAs also significantly affect the proliferation, apoptosis, and invasion of bladder cancer cells. Generally, abnormally expressed circRNAs contribute to the development of bladder cancer primarily by acting as miRNA sponges, which promotes cell proliferation and invasion through mechanisms such as cell cycle regulation, impacts on cell viability, and epithelial-mesenchymal transition (101–114). Additionally, several circRNAs have been investigated as potential biomarkers for bladder cancer prediction. Notably, some overexpressed circRNAs, including circLRIG1, circSTK39, and circITGA7, have shown a significant ability to inhibit bladder cancer growth and metastasis *in vivo* and ex vivo, suggesting they may have a protective function (115–117). In the context of tumor immunity regulation, circZNF609 has been identified as a factor that diminishes the efficacy of immunotherapy for bladder cancer. This occurs through the upregulation of IGF2BP2, which facilitates immune escape of bladder cancer cells (118). Similarly, research indicates that circLOC729852 affects autophagy signaling in bladder cancer cells and augments the recruitment and M2 polarization of tumor-associated macrophages, thereby promoting further tumor growth (119). Conversely, increased expression of circRNA_0013936 has been reported to suppress bladder cancer cells by activating JAK2 and CREB1. This activation promotes the production of immunosuppressive cytokines and enhances anti-tumor immunity (120). Additionally, several circRNAs, including circATIC, circZNF609, and circ_104797, have been demonstrated to increase resistance to cisplatin and promote further progression of bladder cancer (103, 105, 121). Unlike the above two types of RNA which directly target bladder cancer-related genes, research on circRNA in bladder cancer mainly centers on its role as a miRNA sponge. Abnormally expressed circRNAs promote the occurrence of bladder cancer through this mechanism, such as promoting cell proliferation and invasion. However, there are also some circRNAs that exhibit inhibitory effects on bladder cancer. Additionally, some circRNAs also demonstrate the ability to regulate the anti-tumor immunity of the host, which exerts an important influence on the regulation of tumor immune resistance and treatment.

**Table 3:** Overview of circRNA functions in bladder cancer.

lncRNA	Expression	Function	Target miRNA	MiRNA target genes	Reference
circPSMA7	Up	Proliferation (+) Metastasis (+)	miR-128-3p	MAPK1	(102)
circATIC	Up	Proliferation (+) Metastasis (+) Resistance (+)	miR-1247-5p	RCC2	(103)
Hsa_circ_0001583	Up	Metastasis (+)	miR-126-3p	SND1	(104)
circLRIG1	Up	Proliferation (−) Metastasis (−) Apoptosis (+)	miR-214-3p	LRIG1	(115)
circSTK39/has_circ_0001079	Down	Proliferation (+) Metastasis (+)	miR-135a-5p	NR3C2	(116)
circZNF609	Up	Proliferation (+) Metastasis (+) Resistance (+)	miR-1200	CDC25B	(105)
circRAPGEF5	Up	Proliferation (+) Metastasis (+)	miR-582-3p	KIF3A	(106)
circFSCN1	Up	Proliferation (+) Metastasis (+)	miR-145-5p	MDM2	(107)
circLOC729852	Up	Proliferation (+) Metastasis (+) Immunosuppression (+)	miR-769-5p	IL-10	(119)
circRNA CCT3	Up	Proliferation (+)	miR-135a-5p	PP2A	(108)
circTAF15	Down	Proliferation (−) Metastasis (−) Glycolysis(−)	miR-502-5p	HMGB3	(109)
circITGA7	Up	Proliferation (−) Metastasis (−)	miR-330-3p	KLF10	(117)
circMCTP2	Down	Proliferation (−) Metastasis (−) Apoptosis (+)	circMCTP2	FZD8	(110)
circRNA_0013936	Up	Immunosuppression (+)	miR-320a	JAK2	(120)
circRNA_0013936	Up	Immunosuppression (+)	miR-301b-3p	CREB1	(120)
circSTX6/hsa_circ_0007905	Up	Metastasis (+)	miR-515-3p	SUZ12	(111)
circNIPBL/hsa_circ_0001472	Up	Metastasis (+)	miR-16-2-3p	Wnt5a	(112)
circ_104797	Up	Resistance (+)	miR-103a and miR-660-3p	Not mentioned	(121)
circZNF609	Up	Immunosuppression (+)	Not mentioned	IGF2BP2	(118)
circUGGT2	Up	Metastasis (+)	Not mentioned	KU70 and KU80	(113)
hsa_circ_0005320	Up	Proliferation (−)	Not mentioned	IGF2BP3	(114)

' + ' indicates the awareness of ' promotion ', ' - ' indicates the awareness of ' inhibition '.

## 4 Bi−direction effects between microbiome and ncRNAs in tumor

As previously elaborated, despite substantial evidence indicating significant differential expression of the GM and ncRNAs in bladder cancer, several questions remain unanswered. These encompass the specific mechanisms by which the GM influences tumor development and the signals that trigger ncRNA alterations during bladder cancer progression. In other words, the mechanisms underlying the interaction between the GM and ncRNAs in bladder cancer necessitate further investigation. Epigenetic regulation is posited as a potential mechanism by which the GM may alter host pathophysiological processes, through various means including non-covalent epigenetic processes that modulate gene expression (122). As previously outlined, a complex and nuanced regulatory network exists between the host and the microbiota, and elucidating this intricate network is a key objective of our future research. Consequently, this review synthesizes the interactions of various non-coding RNAs with the GM and examines the influence of these interactions on tumor development and therapy.

### 4.1 miRNA-gut microbiota interactions in carcinogenesis

There is accumulating evidence that microRNA plays a crucial role in host-microbiota interactions that govern gut health. MicroRNA is increasingly regarded as a key molecule in microbiota-carcinogenesis interactions (123, 124). Initial evidence of miRNA interactions with gut flora originated from a study on Arabidopsis thaliana’s resistance to *Pseudomonas syringae*. Navarro et al. (125) discovered that Arabidopsis recognition of a flagellin-derived peptide from *Pseudomonas syringae* induces miR-393a transcription. This miRNA inhibits the growth hormone receptor, a negative regulator of the plant immune system, ultimately strengthening plant resistance to Pseudomonas syringae infection. Further studies have demonstrated that miRNA and the GM exhibit related changes in the development of human diseases, particularly in tumor-related diseases. For example, a high-starch diet or a high-fat diet can influence the level of some miRNAs while altering the specific microbial composition of the intestine, thereby potentially realizing tumor prevention or promotion (126, 127). With the deepening of research, an increasing amount of evidence (128) shows that miRNA serves as a tool for the GM and plays a role in tumor regulation.

The microbiome has been demonstrated to be associated with various types of cancer, including colorectal, gastric, hepatocellular, and pancreatic cancers (129, 130). It influences carcinogenesis via its impact on metabolism, cell proliferation, inflammation, and immunity (131, 132). Therefore, further elucidation of the interaction between the host microbiome and microRNAs is crucial for regulating the phenotypic role of bladder cancer. Regrettably, although numerous studies have demonstrated that there is a close relationship between the GM, microRNAs, and bladder cancer, there remains a dearth of direct evidence that GM affects bladder cancer through microRNAs. Therefore, we herein summarize the crucial role of the GM in a variety of tumor-related phenotypes via microRNAs. These phenotypes have been demonstrated to be closely related to the occurrence and development of bladder cancer. Consequently, we aim to provide potential evidence of microRNA bridges for the regulation of bladder cancer by the GM, and also hope to attract increased interest from researchers in this research.

Long-term bladder irritants, such as bladder stones and tuberculosis infections, can trigger a localized chronic inflammatory response in the bladder, inducing bladder epithelial cells to release various inflammatory mediators, such as interleukins and tumor necrosis factor, and attracting a variety of immune cells to infiltrate bladder tissue (22). However, these immune cells further release additional inflammatory factors, establishing a persistent chronic inflammatory environment. This inflammatory environment provides favorable conditions for bladder tumorigenesis. On the one hand, inflammatory factors can activate signaling pathways such as NF-κB, resulting in the abnormal expression of cell cycle regulatory proteins and thereby accelerating cell proliferation rates. Simultaneously, they suppress apoptosis, enabling the abnormal proliferation of cells to survive and accumulate (10). On the other hand, chronic inflammation can induce the proliferation and activation of regulatory T cells (Tregs). Tregs induce the formation of an immunosuppressive microenvironment through the secretion of immunosuppressive cytokines, such as IL-10 and TGF-β, thereby facilitating the escape of tumor cells from immune system surveillance and attack and promoting the development of bladder cancer (133). In addition, the inflammatory response can also induce the polarization of tumor-associated macrophages into M2-type macrophages. M2-type macrophages can promote tumor angiogenesis and inhibit the immune response through the secretion of various growth factors and cytokines, thereby offering favorable conditions for the growth and metastasis of tumor cells (134).

Since the discovery of the GM, it has been identified as having a close association with the immune system. This is manifested, on one hand, in its influence on host immune development and, on the other hand, in its modulation of host immunity. Early in life, colonization of the GM can assist the neonatal immune system in differentiating between self and non-self antigens and establishing immune tolerance (135). It can also exert an influence on the development and maturation of systemic immune organs such as the spleen and thymus, further regulating the overall activity of the immune system (136). In regulating host immune function, the GM can enhance intrinsic immune defense by activating Toll-like receptors and the complement system (137). It also regulates adaptive immune responses by influencing the function of intestinal epithelial cells and dendritic cells (DCs), as well as regulating antibody production and immune memory of B cells (138). These effects are crucial for the regulation of the host inflammatory response. Although the importance of the gut microbiome in the immune-inflammatory response is well established, the exact mechanism of action is still being explored.

**Figure 1:**
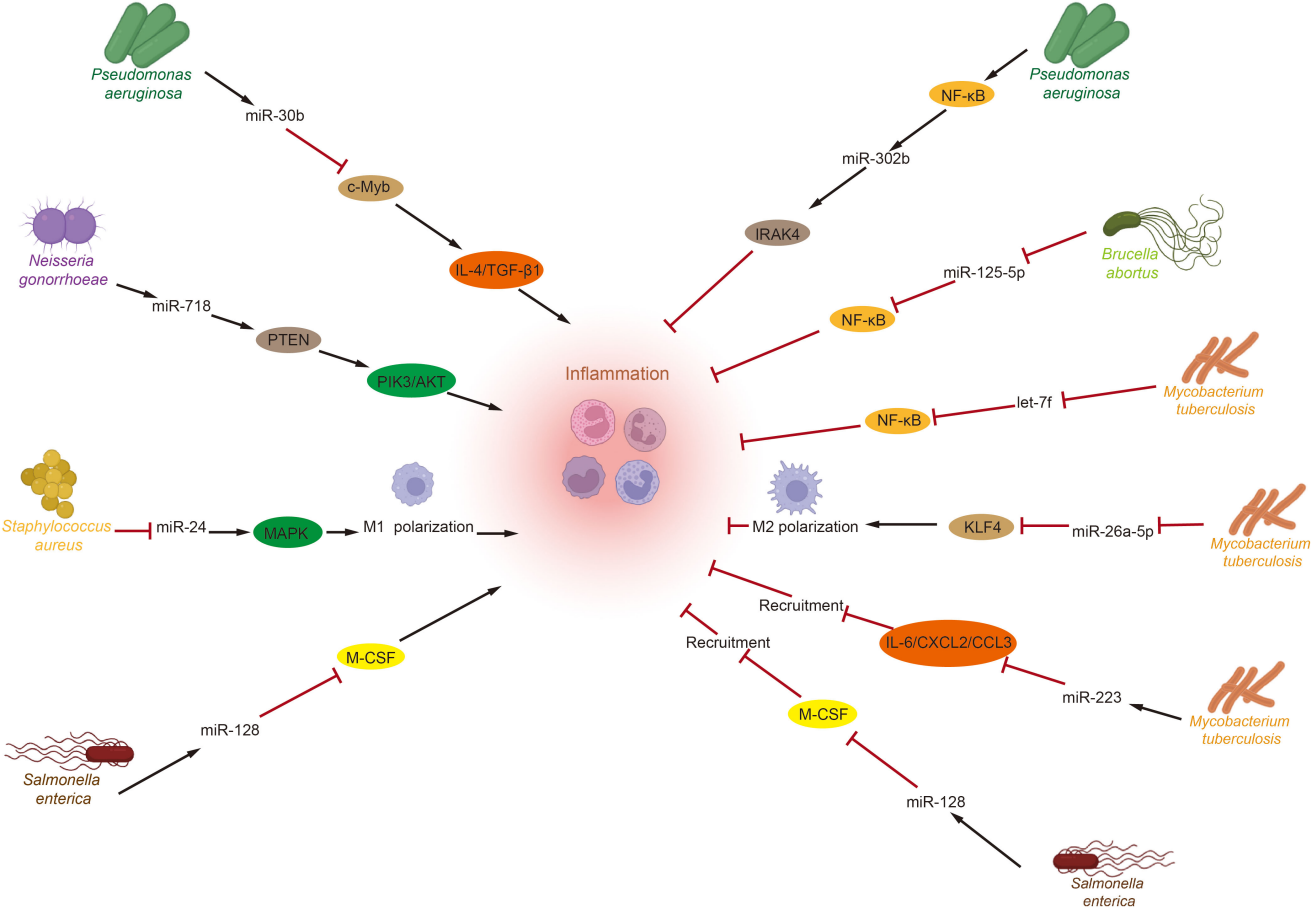
Crosstalk between gut microbiota and miRNAs in inflammation. After Toll-like receptors recognize pathogen-associated molecular patterns from various intestinal microorganisms, several miRNAs related to inflammation regulation are activated. These miRNAs then perform their functions by targeting specific genes, influencing pathways associated with inflammatory responses, regulating macrophage polarization, and modulating the recruitment of inflammatory cells to either promote or inhibit the progression of inflammation. (IL, Interleukin; TGF-β1, transforming growth factor-β1; PTEN, phosphatase and tensin homolog; PI3K, phosphatidylinositide 3-kinase; AKT, protein kinase B; MAPK, mitogen-activated protein kinases; M-CSF, macrophage colony-stimulating factor; IRAK4, interleukin-1 receptor-associated kinase 4; KLF4, kruppel-like factor 4; CXCL2, C-X-C motif chemokine 2; CCL3, C-C motif chemokine ligand 3).

Recent studies emphasize the significant roles of the GM and miRNAs in regulating immune inflammation (**Figure 1**). Toll-like receptors (TLRs) that recognize pathogen-associated molecular patterns (PAMPs) from various microorganisms activate numerous microRNAs that modulate inflammation levels, including miR-146a/b, miR-155, miR-21, and let-7. These microRNAs are known to play crucial roles in balancing the host’s inflammatory response (139–141). *Pseudomonas aeruginosa* infection induces miR-302b expression via a TLR/NF-κB-dependent pathway. miR-302b targets IRAK4, mitigating inflammatory exacerbation by inhibiting TLR-induced NF-κB activation (142). These findings indicate that microRNAs are novel regulators of inflammation, providing negative feedback to TLR-mediated immunity. Additionally, increased miR-301b expression following *Pseudomonas aeruginosa* infection has been shown to modulate inflammation. miR-301b targets c-Myb, decreasing anti-inflammatory factors IL-4 and TGF-β1, which exacerbates the inflammatory response (143). *Neisseria gonorrhoeae* also modulates PI3K/AKT signaling by directly targeting phosphatase and tensin homolog (PTEN) via miR-718, suppressing pro-inflammatory factor production (144). Furthermore, *Brucella abortus* and *Mycobacterium tuberculosis* inhibit the inflammatory response to enhance their survival by downregulating NF-κB pathway levels via miR-125b-5p and let-7f, respectively (145, 146). Besides influencing inflammatory factors, the GM also affects inflammation progression by altering macrophage polarization through microRNAs. For example, *Mycobacterium tuberculosis* infection reduces miR-26a-5p, decreasing kruppel—like factor 4 (KLF4). And promoting M2 macrophage polarization (147). While *Staphylococcus aureus* infection enhances M1 polarization by lowering miR-24, which activates the chi3l1-mediated MAPK pathway (148). Lastly, the GM and microRNAs influence the immune-inflammatory response by modulating inflammatory cell recruitment. *Salmonella enterica* infection increases miR-128 levels, which inhibits macrophage colony-stimulating factor (M-CSF) aggregation at infection sites by directly targeting it (149). Similarly, during *Mycobacterium tuberculosis* infection, upregulation of miR-223 reduces the expression of cytokine IL-6 and chemokines C-X-C motif chemokine 2 (CXCL2) and C-C motif chemokine ligand 3 (CCL3), inhibiting proper neutrophil recruitment to infected tissues (150).

**Figure 2:**
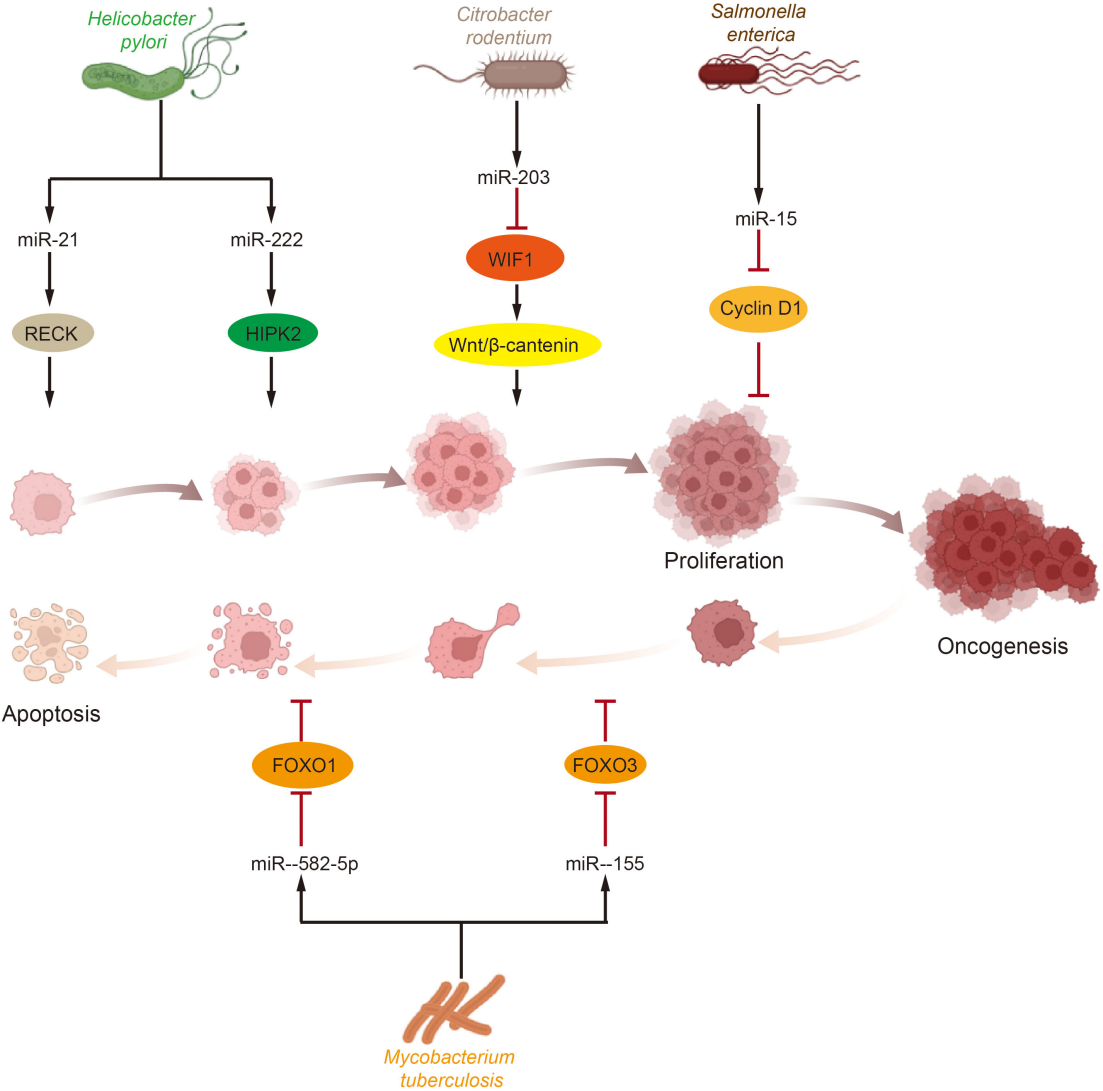
Effects of gut microbiota and miRNA on tumor cell proliferation and apoptosis. Tumor cells proliferation and apoptosis are crucial processes influencing tumorigenesis and development and are significant targets for microbial intervention. Various intestinal microorganisms impact key molecules or pathways involved in tumor survival by modulating miRNA levels, which in turn affect tumor cell proliferation and apoptosis. (HIPK2, homeodomain interacting protein kinase 2; WIF1, Wnt inhibitory factor-1; FOXO, forkhead box O).

Abnormal levels of cell proliferation and apoptosis are the most crucial factors influencing tumorigenesis. In bladder cancer tissues, abnormal levels of certain growth factors and signaling pathways were detected to be abnormally activated, and dysregulated levels of cell cycle protein expression were observed (151). Simultaneously, the regulation of cell proliferation and apoptosis is also a key target for microbial intervention, with *Helicobacter pylori* infection serving as a notable example. Several microRNAs, including miR-203, miR-204, miR-212-3p, miR-320, miR-361-3p, miR-375, miR-584, and miR-1290, have been implicated in *Helicobacter pylori*-dependent cell proliferation and tumorigenesis (152–157). Further research has shown that *Helicobacter pylori* infection elevates the levels of miR-21 and miR-222 in cells and tissues. These microRNAs target tumor suppressor RECK and protein kinase homeodomain interacting protein kinase 2 (HIPK2), which interacts with the RECK homology domain, thereby promoting tumor cell proliferation and invasion (158–160). This body of research suggests that microRNAs upregulated during *Helicobacter pylori* infection play a significant role in promoting tumor cell proliferation and development. Beyond *Helicobacter pylori*, other bacterial microorganisms have also been reported to influence host cell proliferation via microRNAs. For example, *Salmonella* infection reduces the level of miR-15, thereby inhibiting Cyclin D1 and blocking cell cycle progression (161). Additionally, Citrobacter rodentium infection increases the level of miR-203 and stimulates cell proliferation by counteracting the inhibitory effects of Wnt inhibitory factor-1 (WIF1) on the Wnt/β-catenin pathway (162). Moreover, microRNAs regulated by the GM have been shown to participate in cell death pathways. For instance, miR-582-5p and miR-155, which are upregulated following *Mycobacterium tuberculosis* infection, reduce apoptosis by decreasing the levels of the transcription factors forkhead box O 1 (FOXO1) and forkhead box O 3 (FOXO3), respectively (163, 164) (**Figure 2**).

Autophagy, regarded as type II programmed cell death, is a cellular digestive process primarily accountable for the degradation of damaged, denatured, or senescent macromolecules and organelles through lysosomes (165). It also plays a vital role in the host’s innate immune response. Like most tumors, the role of autophagy in bladder cancer is often dual. On the one hand, autophagy assists bladder cancer tumor cells in maintaining cell survival by degrading their own cytoplasmic components, such as proteins and organelles, to supply energy and metabolic substrates for the cells. Meanwhile, an elevated autophagy level also augments the invasive and metastatic ability of tumor cells by regulating the EMT process of tumor cells (166). Conversely, autophagy also exerts an inhibitory role in bladder cancer. For instance, autophagy can promptly eliminate damaged organelles and misfolded proteins from tumor cells, reduce intracellular oxidative stress and DNA damage, and thereby prevent genomic instability (167). Meanwhile, autophagy can also facilitate protein degradation and antigen presentation in tumor cells, enhancing the tumor immune response. For instance, autophagy can promote the maturation and antigen-presenting ability of DCs and enhance the activation and proliferation of T cells (168).

**Figure 3:**
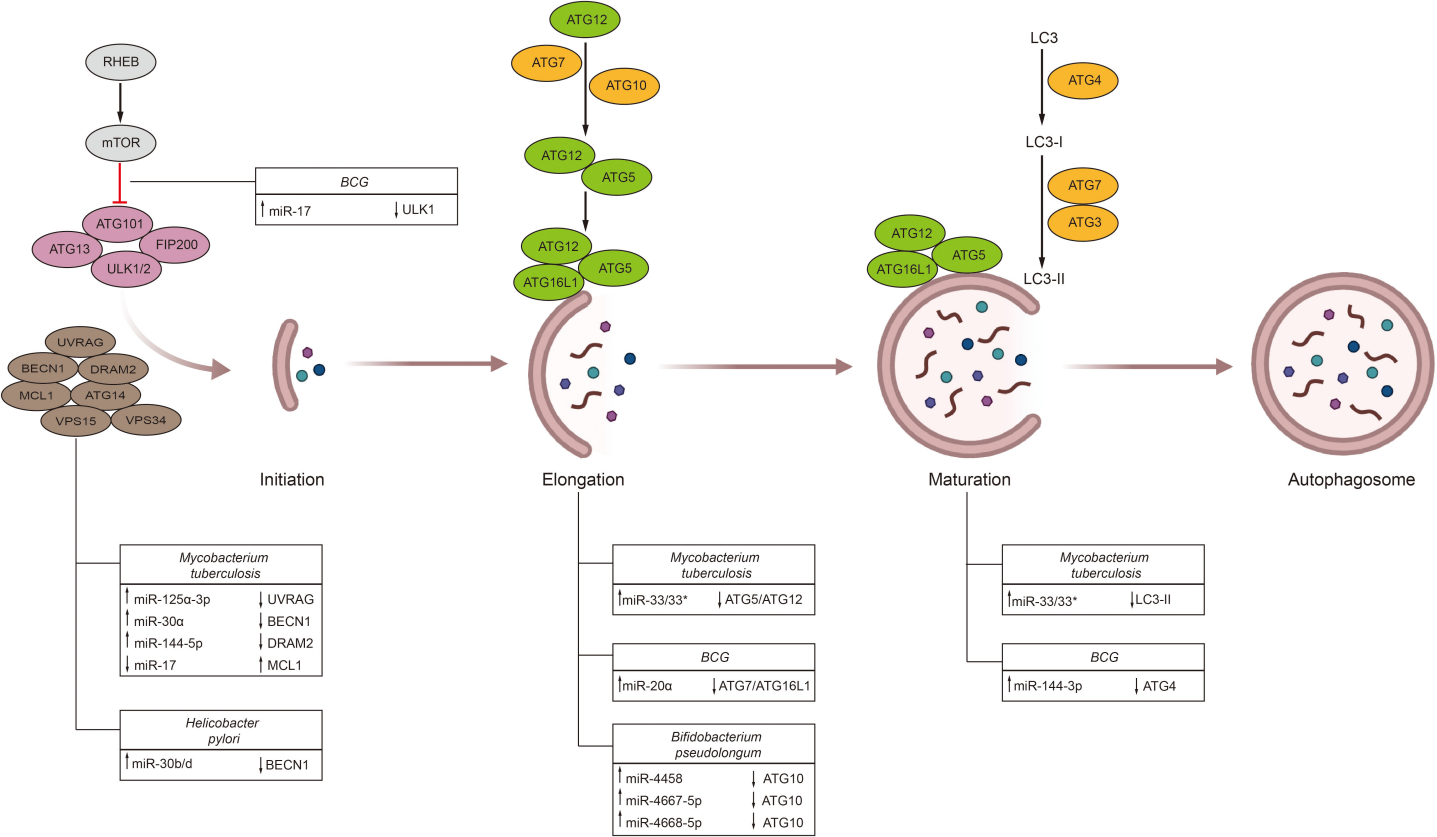
Influence of gut microbiota on the process of host autophagy. Autophagy is a crucial process influencing tumor cell survival and drug resistance. Additionally, bacterial microorganisms have evolved various strategies to modulate the host autophagy process through miRNAs. These miRNAs intervene at multiple stages of autophagy, including initiation, elongation, and maturation. (RHEB, ras homology enriched in brain; mTOR, mammalian target of rapamycin; ATG, autophagy-related gene; FIP, focal adhesion kinase family interacting protein; ULK, unc-51 like autophagy activating kinase 1; UVRAG, UV radiation resistance associated; BECN1, Beclin1; DRAM2, DNA damage regulated autophagy modulator 2; MCL1, myeloid cell leukemia 1; VPS, vacuolar protein sorting; LC3, light chain 3).

Remarkably, bacterial microorganisms have evolved diverse strategies to counter autophagy, including the regulation of host autophagy via microRNAs (**Figure 3**). For example, different mycobacterial species, such as *Mycobacterium tuberculosis* and *Mycobacterium bovis Bacillus Calmette-Guérin* (BCG), modulate host microRNAs to inhibit autophagy. *Mycobacterium tuberculosis* infection augments bacterial survival by inhibiting the autophagy regulator myeloid cell leukemia 1 (MCL1) via reduced levels of miR-17, thereby diminishing autophagy in host cells (169). Similarly, *Mycobacterium tuberculosis* can suppress autophagy by targeting the regulator DNA damage regulated autophagy modulator 2 (DRAM2) via upregulation of miR-144-5p. Moreover, Mycobacterium tuberculosis elevates the expression of miR-30a and miR-125a-3p, which disrupt the initial steps of autophagy by inhibiting Beclin-1 and UV radiation resistance associated (UVRAG), respectively (170, 171). Furthermore, *Mycobacterium tuberculosis* induces miR-33 and miR-33*, which target multiple autophagy and lysosomal effectors (including ATG5, ATG12, LC3B, LAMP1) (172). While microRNAs generally undermine host autophagy to promote mycobacterial survival, *Mycobacterium tuberculosis* has also been reported to enhance autophagy via miR-26a (147). On the other hand, BCG infection induces miR-17 expression and inhibits the autophagy initiation protein unc-51 like autophagy activating kinase 1 (ULK1) (173). Furthermore, BCG suppresses autophagosome formation by upregulating miR-20a and miR-144-3p, which inhibit ATG7, ATG16L1, and ATG4a (174, 175). The regulation of autophagy by microRNAs has also been observed in other microorganisms. For instance, *Helicobacter pylori* infection upregulates miR-30b, leading to reduced levels of Beclin-1 and ATG12, thereby inhibiting autophagy and preventing bacterial clearance (176). Similarly, *Burkholderia pseudomallei* infection in lung epithelial cells elevates the expression of miR-4458, miR-4667-5p, and miR-4668-5p, which inhibit autophagy and promote bacterial survival by suppressing ATG10 expression (177).

The aforementioned studies demonstrate that microRNAs can act as effector molecules of the GM involved in the phenotypic regulation related to bladder cancer. However, it is also shown that the microbial composition in the gut is influenced by microRNAs. Liu et al. (178) identified intestinal epithelial cells and Hopx-positive cells as the primary sources of fecal microRNAs. These microRNAs can regulate bacterial gene expression and affect microbial growth upon entering bacteria. Notably, they discovered that hsa-miR-515-5p and hsa-miR-1226-5p promote the growth of *Clostridium nucleatum* and *Escherichia coli*, respectively, thereby contributing to colorectal carcinogenesis. Additionally, their research (179) revealed that exogenous microRNAs can regulate intestinal bacterial abundance. Specifically, orally administered miR-30d modulates lactase expression in *Akkermansia muciniphila*, further enhancing its abundance in the intestinal tract. Teng et al. (180). demonstrated that exosomal microRNAs from plant sources can affect the intestinal microbiota. Their study showed that exosome-like nanoparticles (ELNs) from edible plants are preferentially absorbed by intestinal bacteria through an ELN lipid-dependent mechanism. Moreover, the extracellular vesicle form of microRNAs can persist in the digestive system, targeting gut microorganisms and strengthening intestinal barrier function. These findings are promising and suggest that therapeutic microRNAs could be extracted from healthy human feces and encapsulated in exosomes to modulate the intestinal microbiota more precisely, potentially preventing disease onset. Additionally, microRNAs might influence bacterial abundance in the tumor microenvironment by regulating glucose metabolism. Yuan et al. (181) observed that bacteria such as cytosolic pseudopods could be regulated by increased polysaccharide production associated with microRNAs, highlighting a potential mechanism by which microRNAs modulate microbial composition through glucose metabolism.

### 4.2 Interaction of circRNA, lncRNA and gut microbiota in tumors

Several studies have reported a close relationship between lncRNAs and the GM. Zhou et al. (182) compared the expression profiles of lncRNAs in the hippocampus of Specific Pathogen-Free (SPF) and Germ-Free (GF) mice, identifying 2,230 differentially expressed lncRNAs (1,355 upregulated and 875 downregulated). This study was the first to report the influence of the GM on hippocampal lncRNAs. Similarly, Zhang et al. (183) found that intramuscular injection of Esketamine significantly changed the GM in mice, including *Adlercreutzia equolifaciens* and *Akkermansia muciniphila*. Additionally, gene expression analysis revealed that 6 lncRNAs were significantly upregulated following Esketamine injection. Several studies have confirmed that the GM can affect host physiological functions by modulating lncRNAs. For example, a recent study demonstrated that the GM could recode lipid metabolism in the intestine via long-chain non-coding RNA Snhg9 (184). Furthermore, another study (185) found that oral administration of *Bacillus fragilis* enhanced lncRNA-CGB expression and promoted anti-tuberculosis immunity. This research also revealed that lncRNA-CGB interacts with EZH2, negatively regulating the epigenetic programming of H3K27 trimethylation (H3K27Me3) and enhancing IFN-γ expression, thus highlighting the crucial role of the GM in regulating immune responses. Additionally, a study (186) on preeclampsia indicated that dysregulation of the gut microbiome could affect trophoblast cell proliferation, invasion, and migration by modulating the NF-κB pathway through lncRNA BC030099. Similarly, *Fusobacterium nucleatum* was found to induce IRF5 expression via the lncRNA ENO1-IT1/miR-22-3p axis, exacerbating neonatal necrotizing enterocolitis (187). Additionally, the close relationship between lncRNAs and the GM has been shown to play a similar role in tumors. Stool testing and 16S rRNA sequencing of colorectal cancer patients revealed notable differences in the gut microbiome and lncRNA profiles (188). Further research demonstrated that *Fusobacterium nucleatum* targets the lncRNA ENO1-IT1 to enhance glycolysis and tumorigenesis in colorectal cancer, thereby elucidating the role of the GM in tumor development through lncRNA (189). Although less common, interactions between circRNAs and the GM have been reported. Chen et al. (190) found that circRNAs can change the composition of the intestinal microbiota and affect intestinal homeostasis and physiology in neonatal mice, thereby linking circRNAs with the intestinal microbiome. Furthermore, an additional study (191) has shown that the gut microbiome can inhibit circRNA expression in tumor stem cells in an interleukin-dependent manner and regulate the levels of corresponding microRNAs, which in turn affects lung cancer metastasis. Although less widespread than microRNAs, this at least indicates that lncRNAs and circRNAs are also potential molecules through which the gut microbiome can influence tumorigenesis. Further considering the molecular sponging role of circRNAs, it is reasonable to hypothesize that there is a complex network of interactions between the gut microbiome and non-coding RNAs in the context of bladder cancer waiting to be further explored.

## 5 Conclusion

The relationship between the GM and bladder cancer development is significant. Bladder cancer progression is often associated with deviations of the GM from its normal state. Correcting these deviations can alleviate carcinogen-induced bladder cancer and enhance the efficacy of adjuvant therapy. Additionally, ncRNAs also play a crucial role in bladder cancer development by influencing cell proliferation, apoptosis, invasion, metastasis, and vascular lymphangiogenesis. The GM influences the host’s immune-inflammatory response, as well as cell proliferation, apoptosis, and autophagy, through the modulation of ncRNA levels, which are closely related to bladder cancer development and drug resistance. As effector molecules of intestinal microorganisms, ncRNAs contribute to the epigenetic regulation of host genes by the GM, impacting various pathophysiological processes. In addition, the exciting findings (192, 193) that the GM can affect the levels of ncRNAs not only in intestinal epithelial cells but also outside the host intestine, such as in the aorta and hippocampus, further substantiates our conjecture. However, currently, there is no conclusive research on how the GM affects bladder cancer through ncRNAs. Furthermore, dysbiosis has been shown to affect tumorigenesis by altering metabolite levels, such as short-chain fatty acids, tryptophan metabolites, and secondary bile acids (6). NcRNAs can also regulate bacterial gene expression, influencing gut microbial growth and flora abundance. Therefore, supplementing with specific exogenous ncRNAs could potentially correct deviations in gut flora and restore normal host physiology. This suggests a promising new approach: instead of solely relying on fecal transplantation, we could extract and package ncRNAs into exosomes to modulate the GM levels and prevent diseases in a more targeted manner. Further research is needed to explore and validate this approach.
